# Analysis of Households’ E-Waste Awareness, Disposal Behavior, and Estimation of Potential Waste Mobile Phones towards an Effective E-Waste Management System in Dubai

**DOI:** 10.3390/toxics9100236

**Published:** 2021-09-25

**Authors:** Yousra Attia, Prashant Kumar Soori, Fadi Ghaith

**Affiliations:** School of Engineering & Physical Sciences, Heriot Watt University, Dubai Knowledge Park, Dubai P.O. Box 38103, United Arab Emirates; ya2002@hw.ac.uk

**Keywords:** Dubai-UAE, e-waste, e-waste management system, households, questionnaire survey, waste mobile phones

## Abstract

During the recent decades, the world has seen ongoing economic and technological development which resulted in the generation of huge volumes of electrical and electronic waste (e-waste). In the Middle East, the United Arab Emirates (UAE) ranks among countries with large e-waste generation due to its consumers’ high spending on electronic devices thereby resulting in a high obsolescence rate in the country. Accordingly, this study aims to analyze the e-waste management and recycling practices in the UAE. It takes Dubai as a case study and conducts a structured questionnaire to analyze households’ awareness, consumption of electronic devices in general and mobile phones in particular, and the disposal behavior of e-waste. Waste mobile phones is taken as a key representative in this study, in which potential waste mobile phones is estimated using the Approximation 1 method in the period 2021–2030. Results from the survey illustrated gaps among households’ awareness and disposal behavior of e-waste, where e-waste recycling rates were noticed to be low. Based on these gaps, strategies were proposed for an effective e-waste management system in the context of Dubai, and were supported by the proposal of an e-waste legislation framework in the UAE.

## 1. Introduction

Electrical and electronic equipment (EEE) has become a necessity with a rapid expansion all over the world, and the increasing demand for these devices has substantially contributed to the generation of large quantities of discarded electrical and electronic equipment.

The ongoing technological developments have shortened the lifespan of EEE products and further increased the number of discarded devices at their end of life (EoL) according to [1,2]. EEE products at their EoL are hence considered as electrical and electronic waste, also known as e-waste [1]. 

E-waste is categorized into six main categories of large equipment, small equipment, screens and monitors, IT or telecommunication devices, temperature exchange equipments, and lamps [3]. 

According to [3], some literature studies have also included leisure and sport equipment, toys, automatic dispensers, and medical devices, as e-waste, however “these equipment are no longer included in the European Union Directive” [3]. 

E-waste has become a global threat due to its increasing large quantities and serious environmental and health issues when handled improperly [4,5]. This is due to the diverse range of hazardous substances contained in e-waste which were found to be more than 1000 and include toxic metals and persistent organic pollutants [6,7,8].

Table 1 provides examples of the common toxic substances reported in e-waste and the serious health impacts associated with them, analyzed by [6,7,9]. 

Moreover, landfilling e-waste generated from different EEE products has negative impacts on the environment. For example, landfilling computer wastes (and many other electronic devices) results in the production of contaminated leachates which in turn leads to groundwater pollution, and melting computer chips produces acids and sludge which lead to soil acidification if disposed of on the ground [10]. According to [11], the cadmium substance found inside one mobile phone battery only is enough to make 600,000 L of water polluted. Incineration of e-waste can also result in the emission of toxic gases which in turn pollute the air [10]. Besides, illegal e-waste recycling produces high levels of air particulate matter (PM10) concentration, and with chronic exposure to air pollution from the informal treatment of e-waste, serious health risks to both humans and animals are created [12]. According to [13], several studies conducted in China showed that, along with human health problems, negative environmental impacts like soil and surface water contamination were significantly associated with the rudimentary recycling equipment used for treating large volumes of e-waste. Besides, the improper e-waste dismantling and burning techniques contribute significantly to air pollution which can cause secondary exposure as pollutants can travel thousands of miles away from the recycling sites to other occupied areas [13]. The informal handling and disposal of e-waste also have risks on the contamination of the food chain as the accumulation of contaminants (resulting from e-waste) in the agricultural lands can be uptake by the livestock [13]. In addition, a study by [13] stated that most toxic chemicals found in e-waste have a slow metabolic rate in animals which in turn may bioaccumulate in their organs thereby affecting food products like eggs. 

However, e-waste is not only a source of toxic substances associated with negative impacts on the environment and human health but also a source of a variety of precious materials which the recycling sector in many countries has a great interest in recovering as they have a high and important economic value. With referring to a study by [14], the valuable materials which the electronic equipment is made of thereby found in e-waste fraction are precious metals such as gold, silver, platinum, etc., base metals such as copper, zinc, nickel, etc., and rare earth minerals such as cerium, neodymium, yttrium, etc. The study by [5] considers these materials as valuable for both the industry and the recycling companies which can help in minimizing the depletion of natural and virgin materials. For example, recycling one million mobile phones can generate and save 24 kg of gold, 9000 kg of copper, 250 kg of silver, and 9 kg of palladium [15].

Besides, the total global gold quantity found in e-waste inventory was estimated to be 300 tons at a value reaching 10.4 billion euros in 2014 according to [15]. 

The appropriate recycling of e-waste has therefore an economic value because valuable materials are recovered and used as a source for secondary material supply as well as for reuse in new devices, which in turn help in minimizing the consumption and excessive use of primary raw materials and improve circular economy [2].

The global e-waste generation reported by [16] was estimated to be 53.6 Mt (million tonnes) in 2019, out of which only 17.4% was officially reported as the collected and properly recycled e-waste. This generation has increased from 44.7 Mt in 2016 and is expected to further increase up to 74.7 Mt by 2030 with an annual growth of approximately 2 Mt [6,16]. 

With referring to a study by [1], it was reported that in 2019, Asia had the highest e-waste magnitudes and generated 24.9 Mt of e-waste, and America came in second place and generated 13.1 Mt. This was followed by Europe with a generation of 12 Mt, while Africa generated 2.9 Mt and Oceania had the least generation of 0.7 Mt.

On the other hand, a study by [16] reported that in 2019, Europe had the highest collection and recycling rate with a percentage of 42.5%, while Asia came at second place with a percentage rate of 11.7%. This was followed by the Americas (9.4%) and the Oceana (8.8%), while Africa had the least percentage rate of 0.9%.

Over the past four decades, in the Gulf Cooperation Council (GCC) region, the economic development with the fast population growth and the rise in the regions’ standard of living has resulted in a substantial growth in the e-waste generation [17]. However, very few literature studies have shed the light on e-waste management in the GCC region. For example, a study by [18] estimated e-waste generation in the GCC region to be 857 kt (kilotonnes) in 2018, with an annual growth rate of e-waste in the range of 3–5%. [18]. 

The main challenges in e-waste management in the GCC region are the lack of e-waste related policies and recycling facilities, the poor or absence of e-waste inventory, and the scarcity in the assessment of e-waste quantities [14,17]. 

With referring to [18] forecasting model from 2018–2040, results have estimated the total e-waste generation in the GCC countries to grow drastically up to 1.094 Mt by 2040 [18]. Therefore, building more e-waste recycling facilities, developing advanced e-waste inventory, and issuing e-waste legislation are becoming a necessity for efficient and good management of e-wastes in the GCC region.

To the authors’ best knowledge, the academic research and literature in the e-waste field are not well explored and very scarce in the UAE. Therefore, the main aim of this study is to examine e-waste management in the UAE in more depth. 

UAE is among countries with a very high obsolescence rate, thereby generating large volumes of e-waste. With reference to the UN statistics, UAE generates 17.2 kg/capita of e-waste annually thereby ranking among countries with the highest e-waste generation in the Middle East [19]. 

UAE is recently giving more attention to the growing concern of e-waste by establishing e-waste companies and facilities specialized in recycling and refurbishing e-waste to further promote effective sustainable waste management activities in the country. Dubai for example has recently made its newest and greatest step in the e-waste field by establishing the world’s largest e-waste recycling plant located at Dubai industrial park, Enviroserve [19]. This 26,012.851 $m^{2}$ e-waste recycling plant features Swiss technology, operates on solar energy, and has a large capacity of 39,000 tonnes of e-waste treatement per year [19,20]. However, no data regarding the amounts of e-waste which have been treated were published by the plant yet. 

In addition to that, UAE is aiming to launch the world’s smartest e-waste bin, designed by Baharash Architecture to coincide with the Dubai Expo 2021, with this e-bin ability to communicate with the users about their discarded electronics recycling compatibility and with the e-waste collection company relevant information about its filling status [19]. 

However, despite the establishment of different e-waste recycling companies in compliance with UAE municipalities around the country, e-waste collection and recycling rates are still low and slowly evolving especially within households, and the UAE still requires more efforts for creating an advanced e-waste inventory and raising awareness among UAE households to establish an advanced e-waste management system. 

With reference to [21], waste in the UAE is classified into the two main categories of non-hazardous and hazardous waste. However, e-waste was not explicitly mentioned in the country’s federal law. A summary of the types of waste stated in the latest federal law No.12 of 2018 on integrated waste management is illustrated in Table 2. 

Due to the lack of research studies on e-waste management in the context of the UAE, this study is conducted to meet the objectives listed below: 

To analyse households’ awareness about e-waste management and recycling, their consumption, and disposal behaviour of discarded electronic devices in general and waste mobile phones in particular via conducting a survey in the context of Dubai, UAE.

To estimate potential waste mobile phones generated by households in Dubai based on the currently in-use mobile phones in 2021, forecast its generation between 2022–2030, analyse results based on a baseline, up, and a low scenario, and study different possible disposal pathways of discarded mobile phones for the 2021 scenario. 

To propose strategies and approaches for an effective e-waste management system in the context of Dubai, UAE.

To propose a framework for e-waste legislation to be adopted by the UAE for effective e-waste management.

## 2. Methodology

The study of e-waste management in the context of the UAE is still evolving and very few literature studies have estimated e-waste generation volumes and analysed the current e-waste recycling strategies and management status for the proposal of a better e-waste management system in the UAE. Therefore, this study served as a good starting point for making further research and advancements in the e-waste field in the UAE. 

Data collection and e-waste management analysis done in this study were regional based and focused on the emirate of Dubai. This can be seen as a good starting point that will contribute to the growing e-waste management efforts currently done by Dubai, and will also help in the analysis of e-waste management in other UAE emirates in future studies.

This study also focused on Dubai households, and the selection of households over other sectors is because households have a very high contribution to EEE products consumption according to [16], thereby adding significantly to e-waste generation volumes. 

E-waste is a vast stream and covers a wide range of EEE categories. This study focused on one widely used electronic device, mobile phones. The selection of mobile phones over other electronics was due to their large product consumption quantity, continuously decreasing lifespan, and high obsolescence rate as a result of the ongoing advancements and new models launched every year, thereby making this device highly discarded by consumers and adding more to the accumulated e-waste in the country on annual basis. UAE also has a high mobile phones penetration rate and is among the highest in the Middle East according to [22]. Hence, the consumption of mobile phones is very high in the UAE, which makes the resulting waste mobile phones add a significant portion to the e-waste stream, thereby requiring more attention. 

**Figure 1:**
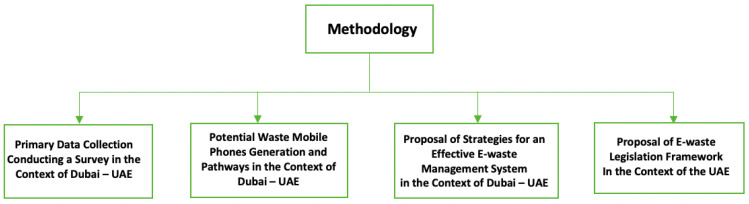
Proposed Methodology Overview.

In summary, this study adopted a specific methodology and covered four main subsections as illustrated in Figure 1. 

### 2.1. Primary Data Collection—Conducting a Survey in the Context of Dubai, UAE

A survey was conducted online on a regional level targeting the households in the emirate of Dubai to analyze their awareness about e-waste, its negative impacts, e-waste recycling practices, as well as their consumption and disposal behavior of e-waste in general and mobile phones as one specific electronics category. 

The survey was only conducted online and for a duration of three weeks. The survey was designed on SurveyMonkey tool [23] and distributed among consumers from different age groups all residing in Dubai. The distribution was based on the snowball sampling among consumers from the author’s contacts, colleagues, family’ friends, colleagues’ friends, and groups which the author is joining, and through a link created by SurveyMonkey tool sent via social media apps and emails. A great percentage of the author’s contacts which the survey was distributed among are young consumers. Therefore, this survey mainly focused on young consumers as they are at the forefront of electronic devices consumption and high obsolescence rate. The survey questions were written in the English language and targeted local residents only. Based on that, a total of 114 reliable responses were collected and analysed in this study. 

Due to its effectiveness and reliability, SurveyMonkey tool was also used to analyse, graph, and illustrate the survey results in bar charts. 

The survey technique is applied on a small audience representing the total population of the targeted location. In this study, it was used to represent a small fraction from Dubai population which has reached 3,438,185 in 2021 [24]. The survey sample size tends to be estimated based on a margin of error, which survey results reliability is based on. The margin of error therefore illustrates the effectiveness of the survey, as the smaller the margin of error is, the better the sample size will be a representative of the population, and by increasing the sample size, the results’ reliability and confidence will further increase [25]. According to [26], sampling results are accurate at any margin of error below 10% at a confidence level of 95%. Therefore, accuracy tends to further increase at even lower margin of error. Equation (1) illustrates the margin of error formula [25].

(1) $$\;\mathrm{Margin}\;\mathrm{of}\;\mathrm{error}=z*\frac{\sigma }{\sqrt{n}}$$

where: z is the z-score, *n* is the survey sample size, and σ is the population standard deviation. 

SurveyMonkey tool also has an effective margin of error calculator which was used to obtain this study survey’s margin of error [25]. Based on the sample size and Dubai population, a margin of error of 9% was obtained at a confidence level of 95%. This margin of error is below 10% which makes the sample size relatively good. However, future studies should increase the sample size for a lower margin of error and a higher confidence as one effective suggestion. 

**Figure 2:**
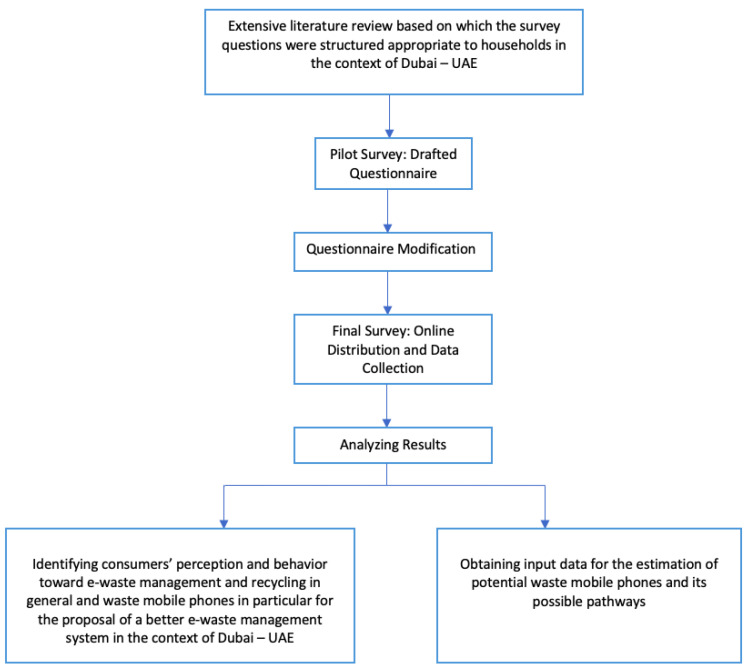
Survey Outline Overview.

A general outline of how the survey was constructed and analysed is summarized in Figure 2.

The survey questions were designed efficiently after an extensive analysis of case studies which used the survey technique to study households’ consumption and disposal behaviour of e-waste such as [27,28,29,30,31,32,33,34].

#### 2.1.1. Survey Overall Design 

The survey questions were first drafted suitable to the study of households in the context of Dubai. After that, a pilot survey was conducted online via social media apps and emails and distributed among 15 random participants from the author’s contacts. These participants were from different age groups, and different genders in order to get more efficient feedbacks. Once the pilot survey was completed, a general feedback and comments were asked to be attached for the author to analyse. The pilot survey was hence conducted to identify any gaps in the survey questions or any ambiguities to be clarified, and to measure the time taken to complete the survey. Based on that, questions were more clarified, simplified, and shortened to keep the time to complete the survey short and efficient, and the final survey was prepared for distribution on a larger sample. 

The final survey questions were 18 multiple-choice questions and were designed into three main sections as summarized in Table 3. 

#### 2.1.2. Survey Results as Input Data to Waste Mobile Phones Estimation 

Primary data collection was used as the best tool for estimating the waste mobile phones through survey conduction. Section 3 questions in the survey were therefore dedicated to mobile phones consumption, active-use, and waste. Results from these questions were used to obtain primary input data for waste mobile phones estimation in Dubai. Further details about the data collection constraints are shown in Section 2.2. 

#### 2.1.3. Survey Results for the Proposal of a Good E-Waste Management System in the Context of Dubai—UAE 

Due to the scarcity of research and literature done on e-waste management in the UAE, this study used results from the survey along with adopting strategies from developed countries to propose good approaches and strategies for an effective e-waste management in Dubai, UAE.

### 2.2. Potential Waste Mobile Phones Generation and Pathways: In the Context of Dubai—UAE

Various methods have been used to estimate e-waste generation from different EEE categories in developing and developed countries, however, each method is used based on the available input data required for the estimation. This input data should be specific to the country of study since each country has different sales scenarios, consumption, and disposal behaviors by its consumers. All these methods are therefore created to suit any country’s e-waste inventory data. In other words, studies about developed countries with advanced e-waste inventory and detailed historical input data tend to estimate e-waste with more detailed e-waste estimation methods, whereas studies on countries with poor e-waste inventory and scarcity in input data use methods that require fewer data for e-waste estimation [35,36,37].

To the authors’ best knowledge, the UAE does not have an advanced e-waste inventory as no official data regarding the breakdown of e-waste generation quantities from different EEE products nor information about e-waste possible pathways and its recycling rates were found or published. 

After an extensive analysis of different e-waste estimation methods in studies such as [31,35,36,37] and their applicability in the context of the UAE, the most suitable method selected for estimating potential waste mobile phones was the Approximation 1 method (also referred to as the Modified Consumption and Use method). This method was selected among other e-waste estimation methods due to the constraints below: 

Historical mobile phones sales data (and related mobile phones imports/exports data) were not found or published in the context of the UAE or Dubai in particular. This was confirmed after contacting and visiting the respective agencies for data procurement in Dubai. 

Historical data about mobile phones stocks, time-series lifespan, waste mobile phones recycling rates, and other waste mobile phones possible pathways rates were not available on both regional and national basis in Dubai and the UAE. 

The collection of primary data via survey was hence selected as the only best alternative, based on which, input data were obtained for the estimation.

Based on the above constraints and limitations, the Approximation 1 method was selected as it does not require historical sales data, and is based on the currently in-use mobile phones (stock) per capita and a fixed average lifespan which can be estimated from the survey results.

The Approximation 1 method is the modified version of the Consumption and Use method. The Consumption and Use method is a common method used in e-waste estimations and is based on the EEE products fixed lifespan duration and the stock level which in turn is based on the number of households and the percentage of households possession of the EEE products [35]. The Approximation 1 method, however, modifies the Consumption and Use method and makes it more accurate and specific by taking the population and the average EEE product consumption per capita for the stock quantity instead of households [36]. The Approximation 1 method is mainly used in countries with scarce input data and not advanced e-waste inventory, similar to the UAE case study. It was used in different studies for estimating e-waste generated from different electronic devices. For example, studies by [31,36] have used the Consumption and Use method and modified it based on the targeted type of the electronic device to estimate e-waste generation from different electronic devices. 

With referring to [36], Equation (2) was therefore used to estimate waste mobile phones in this study.

(2) $$\;\mathrm{WMP}\left(t\right)=\frac{{\mathrm{Stock}}_{\mathrm{private}}\left(t\right)}{L}=\frac{P\left(t\right)*N*W}{L}$$

where: WMP(t) is the waste mobile phones generated in year t, Stock private is the private consumers’ stock of mobile phones, P(t) is the population in year t, N is the average number of mobile phones owned/consumption by a person, W is the average weight of mobile phones, and L represents the average fixed lifespan.

Moreover, secondary data is required for waste mobile phones estimation as summarized in Table 4.

To add more reliability to the estimation and cover the main possible cases of waste mobile phones’ quantities, a sensitivity analysis was carried out and three different scenarios were studied based on the value of the average lifespan used for the estimation as illustrated in Table 5.

Finally, a general Sankey diagram showing the different possible pathways of mobile phones to be potentially discarded in the current year of 2021 was drawn using the e!Sanky tool [41]. Due to the lack of research on the fractions of possible pathways that e-waste can take in Dubai, results obtained from the survey (question 16 results) were used for estimating the possible pathways weights. 

### 2.3. Proposal of Strategies for an Effective E-Waste Management System: In the Context of Dubai—UAE

Based on the results obtained from Section 2.1 and Section 2.2, which supported the assumption of the growing generation pattern of e-waste (through analysing the waste mobile phones as one major e-waste category) and identified gaps in the consumers’ e-waste awareness, strategies were proposed for an effective e-waste management in Dubai. 

### 2.4. Proposal of E-Waste Legislation Framework: In the Context of the UAE 

By analyzing advanced e-waste legislations and policies issued by other developed countries, this study proposed a general framework for e-waste legislation to be adopted by the UAE and hence will assist the UAE government to establish an advanced e-waste management system associated with proper e-waste regulation.

## 3. Results

### 3.1. Primary Data Collection—Survey Results

#### 3.1.1. Sociodemographic Results

Based on the sociodemographic results, 55.3% were females and 44.7% were males. The highest percentage of responses comes from respondents of the age group 18–24 years old with 48.3%. Besides, the highest percentages of 67.5% had bachelor’s degrees, 24.6% had 5 members in their household, and 45.6% had a monthly household income in the range of United Arab Emirates Dirham (AED) 10,001–AED 30,000. 

A detailed summary of the detailed sociodemographic results is illustrated in Table 6.

#### 3.1.2. Awareness about the E-Waste Concept and E-Waste Recycling 

To analyze the consumers’ attitude toward e-waste recycling, it was important to study their perception about the concept of e-waste. When respondents were asked about the terminology/concept of e-waste, only 21.1% believed that they had high awareness, whereas the highest percentage of 33.3% believed that they had no idea, followed by 26.3% who had low awareness about e-waste. Respondents in the age groups 18–24 and 25–29 years old covered a total of 73.7% of the respondents who assured that they had no idea what e-waste is, and this contributes to the highest percentage when compared to other age groups. 

When respondents were asked about their familiarity with e-waste recycling, about 50.9% stated that they were not familiar with it where the majority covered respondents in the age groups 18–24 and 25–29 years old and with a bachelor degree. Besides, when respondents were asked about their awareness of specialized e-waste recycling centers, 77.2% were not aware of any centers with the majority covering the age group of 18–24 years old. 

**Figure 3:**
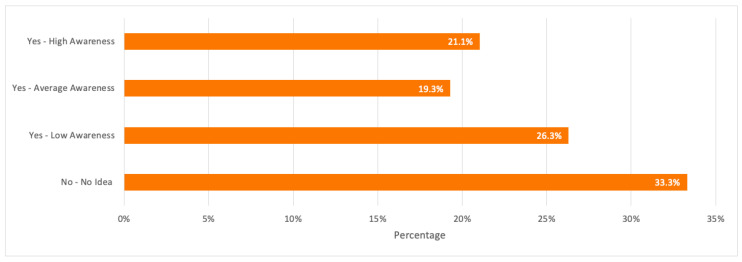
Respondents’ awareness of the concept of electronic waste (e-waste).

**Figure 4:**
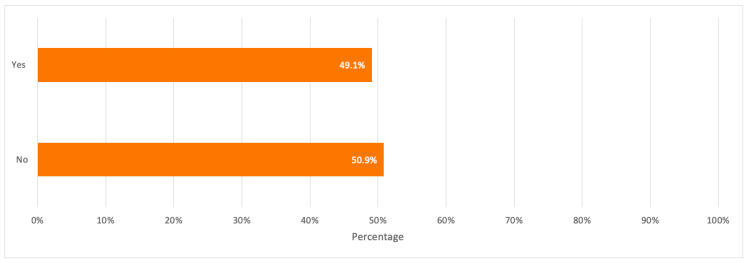
Respondents’ awareness of the practice of e-waste recycling.

**Figure 5:**
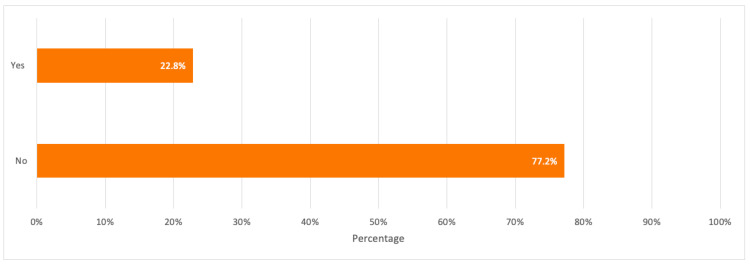
Respondents’ awareness of any recycling companies or centers dedicated to e-waste collection and recycling.

A detailed summary of all the above results is illustrated in the bar charts in Figure 3, Figure 4 and Figure 5.

#### 3.1.3. Participation in Recycling Electronic Devices, Behaviour toward Discarded Devices, and Awareness about E-Waste Toxic Elements’ Negative Impacts 

When respondents were asked about how often they have recycled their electronic devices, the majority with 68.4% have never recycled their devices. It is worth noticing that respondents with high awareness about e-waste contributed the most to recycling their electronic devices more than once with a percentage of 50% when compared to respondents with average to no awareness about e-waste. 

Respondents (whether they have highly participated or not in e-waste recycling) were also asked about their general opinion on the main reason that would make consumers have low to no participation in recycling their discarded devices. The highest percentage of respondents (49.1%) considered the no (or low) awareness on the process of e-waste recycling as the main reason. Besides, when respondents were asked about how they usually deal with their discarded electronic devices by giving laptops, desktops, and tablets as three main examples, 43.0%, which represents the majority of respondents, stated that they store them at home, while 23.7% usually give them away to their friends or family. 

On a positive note, a percentage of 67.5% were aware that the toxic elements found in discarded electronics may have negative impacts on the environment and human health, which reflects the respondents’ good familiarity with pollutants’ negative impacts on the environment. Besides, the majority of respondents showed a positive response to their willingness to participate in e-waste recycling with a percentage of 79.8%. 

**Figure 6:**
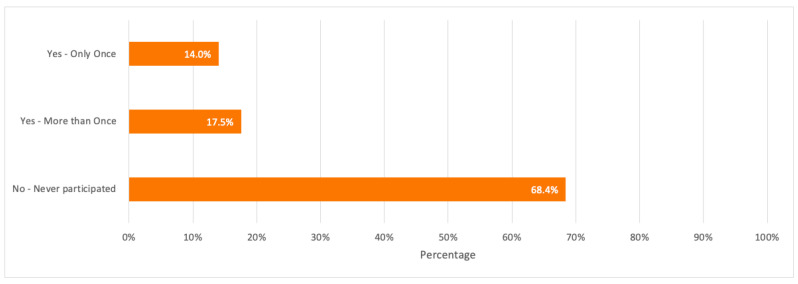
Respondents’ participation in recycling electronic devices.

**Figure 7:**
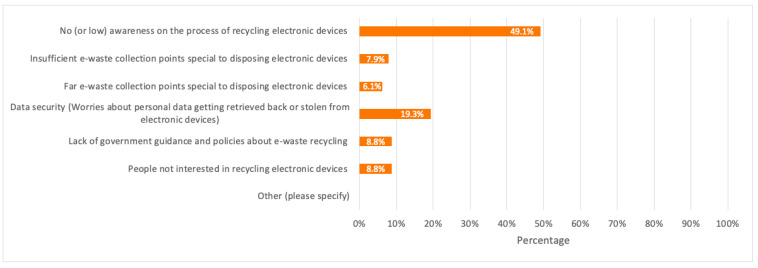
Respondents’ opinion about consumers’ main reason for low or no participation in e-waste recycling.

**Figure 8:**
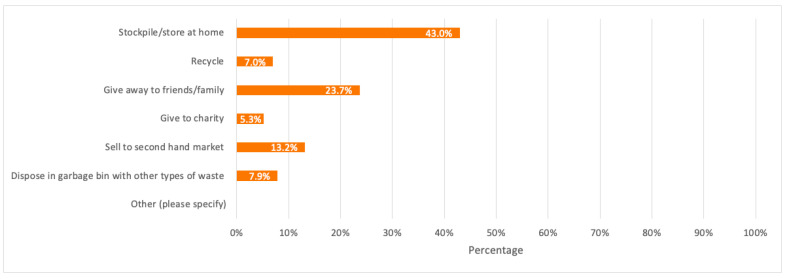
Respondents’ disposal behavior of unused/discarded electronics.

**Figure 9:**
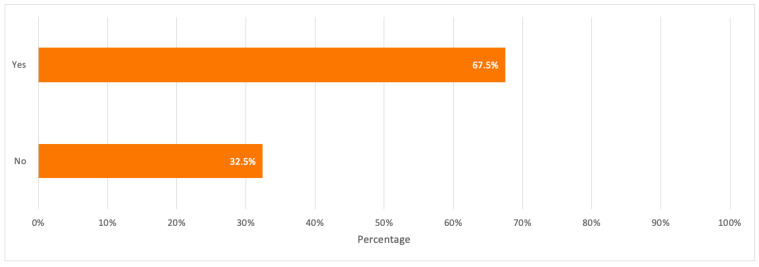
Respondents’ awareness that e-waste toxic elements may be hazardous to the environment and human health.

**Figure 10:**
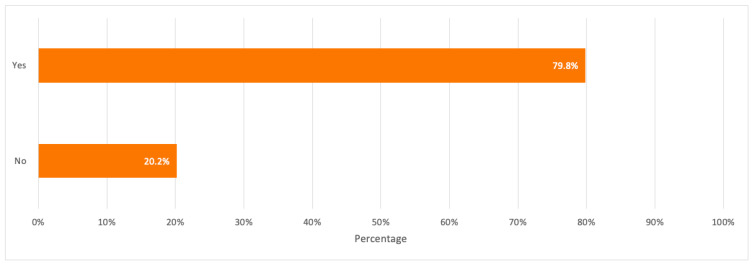
Respondents’ interest/willingness in participating in e-waste recycling.

A detailed summary of all the above results is illustrated in the bar charts in Figure 6, Figure 7, Figure 8, Figure 9 and Figure 10.

#### 3.1.4. Mobile Phones Consumption and Respondents’ Disposal Behaviour toward Discarded Mobile Phones 

**Figure 11:**
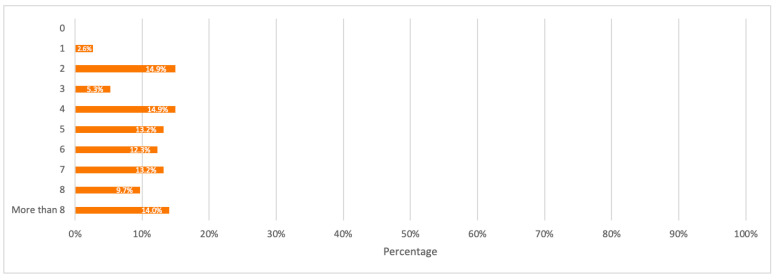
Number of mobile phones currently in-use by households.

Respondents were first asked about the number of mobile phones used in their households. The detailed distribution of mobile phones among respondents is illustrated in Figure 11. Results from this question were used as input data for mobile phones consumption per household and capita. 

They were also asked about the duration of active-use of their mobile phones before they get discarded or changed. Based on the results, the majority use their mobile phones for 2 years (22.8%) to 3 years (29.0%) before they change or discard them. Results from this question were also used as input data for the average useful lifespan of mobile phones for the baseline scenario. 

Respondents were asked again about their disposal behavior but only specific to mobile phones to study the possible pathways of waste mobile phones, where the majority of respondents with 43.0% stated that they store or stockpile their discarded mobile phones at home. 

When asked about the number of old mobile phones stored by households, and for how long respondents tend to store their old mobile phones, the majority of 23.7% have 5 or more mobile phones stored at home, and 32.5% of respondents store their unused mobile phones for over 5 years. 

**Figure 12:**
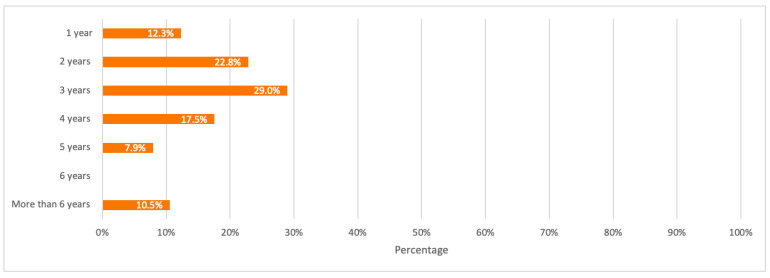
Respondents’ mobile phones active-use duration.

**Figure 13:**
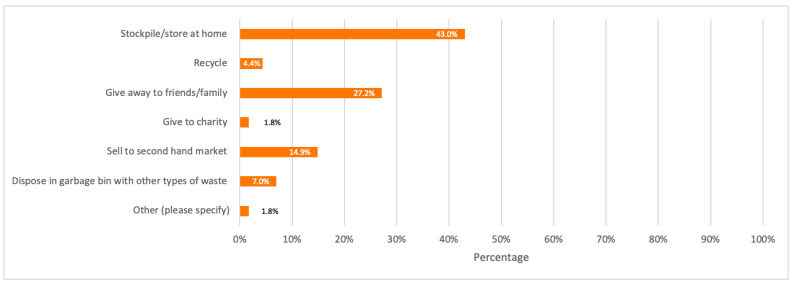
Respondents’ disposal behaviour of not used/old mobile phones.

**Figure 14:**
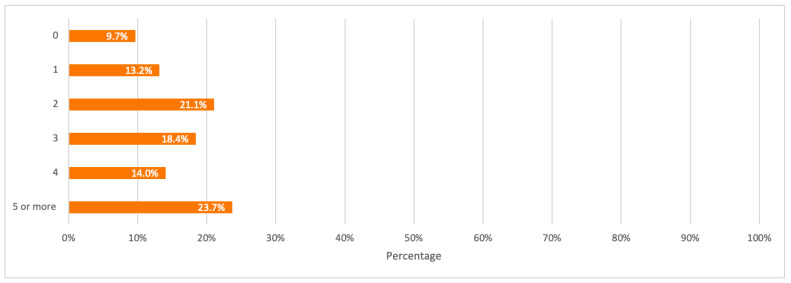
Number of mobile phones not in use and stored in households.

**Figure 15:**
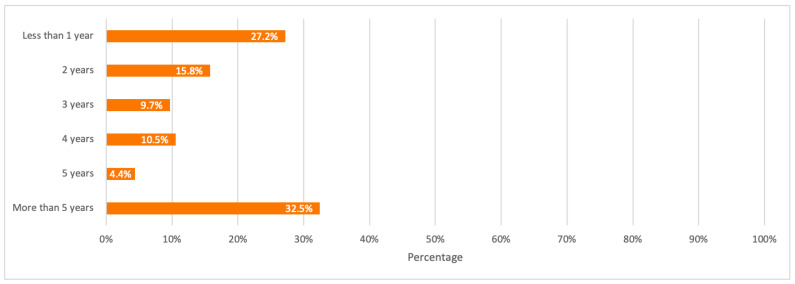
Respondents’ old mobile phones storage duration.

A detailed summary of all the above results is illustrated in the bar charts in Figure 11, Figure 12, Figure 13, Figure 14 and Figure 15.

### 3.2. Extended Survey Results on Households’ Mobile Phones Active-Use Duration vs. Household Monthly Income 

**Figure 16:**
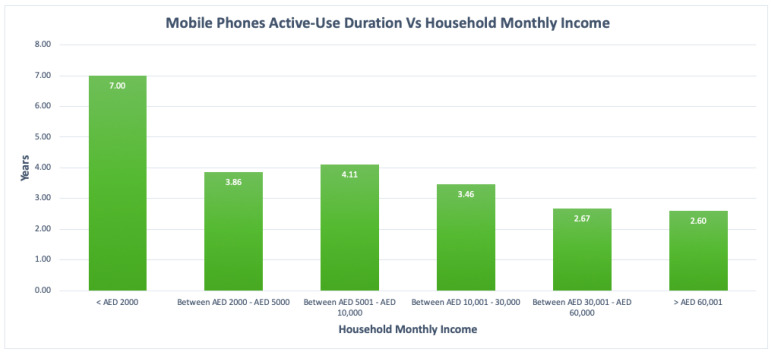
Active-use duration Versus Household monthly income.

To further analyse mobile phones average active-use duration, it was observed that one noticeable important relationship was found between this duration and the households’ monthly income. The average useful lifespan durations of mobile phones were therefore compared among respondents with different household monthly incomes based on the mean values from the survey results. Results are summarized in the bar graph in Figure 16. 

### 3.3. Potential Waste Mobile Phones Generation

To estimate waste mobile phones, values for parameters required in Equation (2) were obtained using both primary and secondary data. Secondary data is summarized earlier in Table 4. 

#### 3.3.1. Primary Data 

Due to e-waste secondary input data limitations in the context of the UAE, the active-use duration and mobile phones current usage (i.e., consumption) were estimated based on the survey results. 

The collected primary data are summarized in Table 7 below. 

Based on the average household size and current average household mobile phones’ consumption, the current average mobile phones’ consumption per capita, which represents the mobile phones stock was estimated using Equation (3).

(3) $$N=Average\;MP\;per\;Person=\frac{Average\;MP_{household}}{Average\;Household\;Size}\;$$

where: *N* = *average MP per person* is the average mobile phones’ consumption per person, and *average* *MP_household_* is the average mobile phones’ consumption per household. 

This gives an average consumption of 1.44 mobile phones/person. The currently in-use mobile phones for Dubai population in 2021 was therefore estimated as 4,950,986 mobile phone units. 

This average value of 1.44 mobile phones per person was assumed based on the survey results since no published data regarding the historical mobile phones stocks were available in the context of Dubai as mentioned earlier. Because consumption or ownership of mobile phones per capita does not get a dramatic change in the short run, this value was assumed not to get affected/unchanged in the targeted forecasted period [16]. 

Lifespan is another important variable for estimating waste mobile phones. Three scenarios were studied and Table 8 summarizes the lifespan durations used in each scenario.

Based on the variables’ values collected from both primary and secondary data and by applying Equation (2), the potential number of mobile phones to be discarded as waste in 2021, based on the baseline scenario, was estimated to be 1,509,447 units or 150.94 tonnes.

#### 3.3.2. Sensitivity Analysis Results 

The potential waste mobile phones were estimated in the period 2021–2030, and Table 9 summarizes the resulted waste mobile phones in the three scenarios by taking the years 2021, 2025, and 2030 as three examples.

**Figure 17:**
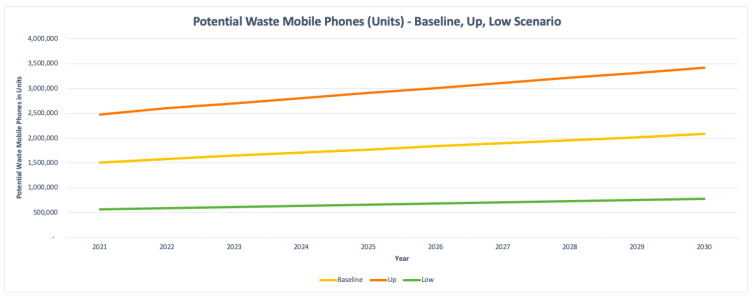
Waste mobile phones—baseline, up, and low scenarios (units).

**Figure 18:**
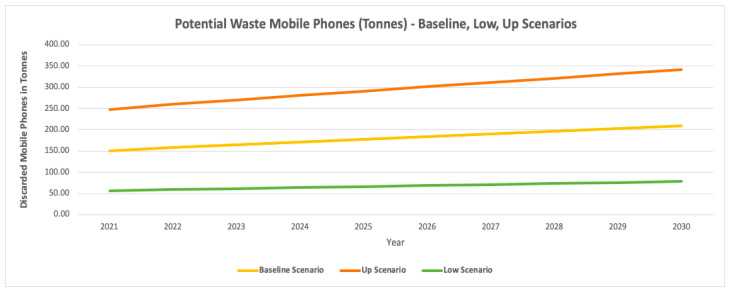
Waste mobile phones—baseline, up, and low scenarios (tonnes).

The potential waste mobile phones are also graphed for the three scenarios in units and tonnes as shown in Figure 17 and Figure 18 respectively. 

#### 3.3.3. Discarded Mobile Phones Possible Pathways—Baseline Scenario 2021

Due to lack of published data, survey results illustrated in Figure 13 were the best alternative to take in order to draw a general Sankey diagram which only shows the possible pathways of discarded mobile phones by their original first owners, and the corresponding weight of each pathway in tonnes, by taking 150.94 tonnes as the amount of decarded mobile phones (as per the baseline scenario) when the mobile phones stock is 495.10 tonnes for Dubai population in 2021. 

To further simplify the possible pathways when drawn on the e!sanky tool, the percentage of respondents who giveaway their discarded mobile phones to family or friends was combined with charity donation. Also, 1.8% of respondents who selected the “others” option stated that they tend to keep their unused mobile phones for themselves for future use if needed. This percentage was combined with the percentage of people who tend to store their mobile phones at home. 

**Figure 19:**
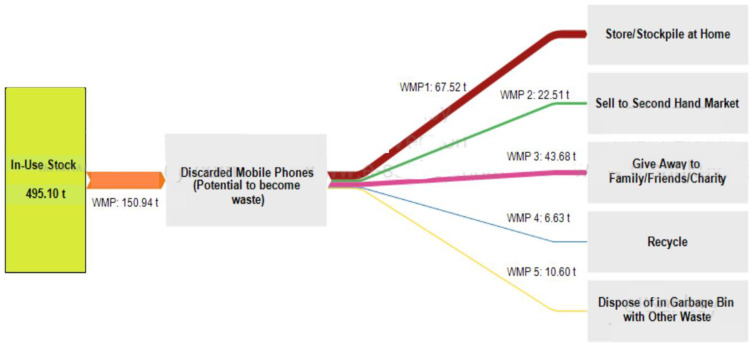
Obsolete/Discarded Mobile Phones Possible Pathways.

The Sankey diagram was drawn using the e!sanky tool as illustrated in Figure 19.

Based on Figure 19, it is observed that the highest discarded mobile phones pathway weight is store/stockpile at home covering 67.52 tonnes, while the lowest pathway weight goes to e-waste recycling covering 6.63 tonnes only.

## 4. Discussion

### 4.1. Proposal of Strategies for an Effective E-Waste Management System in the Context of Dubai, UAE 

From the survey results in Section 3.1, over 50% of respondents had low to no idea about the e-waste concept and e-waste recycling. This reflects a low e-waste awareness for which more e-waste campaigns and awareness programs should be conducted especially among young consumers in schools and universities. A noticeable high percentage of respondents (77.2%) were also not aware of any recycling companies dedicated to e-waste recycling despite having respondents who have recycled their electronic devices at least once (31.6%). This shows that some consumers tend to recycle their devices through collection points or drop-off services without familiarizing themselves with the recycling companies responsible for e-waste collection from these collection points. Therefore, e-waste recycling companies and centres in Dubai should build a public knowledge and familiarize themselves through awareness programs about e-waste, its negative impacts if not properly handled, the importance of e-waste recycling, its process, and provide financial incentives such as gift cards, vouchers, cash, etc. when participating in e-waste recycling as this would help in getting consumers more familiar with these companies, and motivate them to recycle their electronic devices since the majority of the survey respondents (68.4%) have never participated in e-waste recycling. This mass awareness could be created through social media as one effective tool for a faster spread of awareness among consumers. 

Besides, these awareness programs should put attention to data safety and how their recycling processes ensure data wiping from all devices, as this tends to concern consumers and reduce their willingness to recycle, which was also observed in the survey results. 

When it comes to mobile phones, it was observed in the results section that the majority of respondents tend to store their obsolete mobile phones at home, with an average of 3.85 stored mobile phones per household. A similar result was observed in a study by [45] in India with over 50% of respondents in their studies storing their obsolete mobile phones at home. This shows that multiple reasons can make consumers more reluctant to recycling their mobile phones. For example, some consumers tend to have a sentimental attachment to their old mobile phones and give them inherent value, and some others have worries about data security resulting in stockpiling their old mobile phones for many years. 

It is also worth noticing that 7.0% of respondents dispose of their old mobile phones in the garbage with general waste, which shows that a fraction of waste mobile phones (even if small) goes to landfills with other waste, instead of being properly recycled. Based on Figure 13, obsolete mobile phones by 43.9% of respondents are given an extended lifespan by second-hand users for they are either sold to the second-hand market or given away to family, friends, or charity. Therefore, as mentioned earlier, financial incentives can be applied to motivate consumers to extend the lifespan of their working electronic devices and recycle their obsolete not functioning ones instead of stockpiling them at home. 

The average mobile phones active-use duration was further analysed with respect to household monthly income. From Figure 16, and when compared to respondents with higher household income, it was observed that mobile phones are used for longer durations by respondents with low household income. The shortest average useful lifespan was observed to be 2.60 years for respondents with a household income of higher than AED 60,001, which in other words means that households with higher incomes tend to replace their mobile phones more often thereby generating more waste mobile phones. Hence, mobile phones awareness programs should also put a great focus on households with high income as they tend to generate more waste mobile phones.

As one effective suggestion, the promotion of environmental education is necessary for raising e-waste awareness among young consumers, thereby increasing e-waste recycling. A study by [40] has shown that awareness about laptops usage and disposal in Japan had a positive impact on laptops disposal practices and increased their recycling rates. In educational institutions, a green box can be designed as a large container for the disposal of any unused electronic devices to further encourage e-waste recycling by students, faculty, and staff.

For a good e-waste management system, it is thus necessary that a set of stockholders including shared comprehensive efforts from consumers, the Ministry of Climate Change and Environment, the government of Dubai, Dubai municipality, and the importers/retailers of EEE products in Dubai are recognized for e-waste generation from different EEE products as well as their management from the point of purchase till they become obsolete and discarded as e-waste. 

EEE products retailers can contribute to the e-waste recycling by launching recycling programs with collaboration with e-waste recycling companies in Dubai, for disposing of old mobile phones (or any other obsolete devices) by consumers and receiving coupons or gift cards in return to further encourage recycling. 

As per the survey results in Figure 7, a total of 14.0% of respondents believed that insufficient and far e-waste collection points are two main reasons for the no/low participation in e-waste recycling. Dubai already has collection points where consumers can dispose of their e-waste, however, more collection points should be implemented for different EEE categories and close to residential areas. One effective solution is the implementation of curbside recycling bins special for discarding smaller electronic devices such as mobile phones and their accessories next to general waste and plastic curbside bins in households or residential communities. This will further encourage households to dispose of their old small electronic devices into the right bins instead of throwing them with general waste or store them at home. 

In the context of the UAE, an advanced e-waste inventory does not exist, where e-waste estimation and recycling rates are not separated by each EEE category, as per the Ministry of Climate Change and Environment, and does not analyse each category individually. By taking waste mobile phones estimation limitations as one e-waste category example, gaps are justified to be found in the e-waste inventory and management system, which can also justify the scarcity in research studies about e-waste in the context of the UAE. 

Therefore, the UAE government should put more effort into building a comprehensive e-waste inventory covering different e-waste categories. One suggestion can be through the proposal of specialized EEE registers for monitoring and tracking the quantities of e-waste generated from each EEE products categories, e-waste stocks and pathways, and recycling rates on annual basis, thereby controlling the flow of e-waste across the country. 

### 4.2. Potential Waste Mobile Phones Generation and Flow in the Context of Dubai, UAE

In this study, the active-use duration was considered as the average useful lifespan for the baseline scenario. This useful lifespan definition was also used in a study by [46] when they estimated mobile phones lifespan and waste generated in an Australian case study. 

Based on Table 9 results, the estimated waste mobile phones are observing an increase in the coming forecasted years with the population growth in Dubai. For the most realistic baseline scenario, the current (2021) and future potential waste mobile phones by 2030 were estimated to be 150.94 tonnes and 208.53 tonnes respectively. For the up scenario, the current (2021) and future waste mobile phones by 2030 were estimated to be 247.55 tonnes and 341.99 tonnes respectively. However, the low scenario underestimated the waste mobile phones, in which the current (2021) and future waste mobile phones by 2030 were estimated to be 56.65 tonnes and 78.26 tonnes respectively. 

On the other hand, when applying the survey results on the population of Dubai, the possible mobile phones pathways once discarded by consumers and drawn on a Sankey diagram for the current year of 2021 showed that only 6.63 tonnes are recycled from the potential discarded mobile phone of 150.94 tonnes. The recycling rate is therefore considered very low when compared to developed countries with advanced e-waste management systems and relatively higher recycling rates like Switzerland in which digital e-waste (including waste mobile phones) recycling rate reached 95% [47,48]. 

Based on the growing rate of waste mobile phones and the observed relatively low recycling rates, it is concluded that the e-waste recycling companies, in compliance with the Dubai municipality should put more efforts into raising awareness among the population of Dubai about storing less and recycling more obsolete mobile phones for the benefit of the environment and the economy of the country. 

Non-governmental recycling companies and groups in Dubai are contributing to e-waste recycling. However, more efforts should be done for higher collection and recycling rates since waste mobile phones is estimated to have a dramatic increase especially with the future higher penetration rates and population growth in Dubai. 

Overall, gaps exist among consumers’ awareness of e-waste, its recycling, the consumption and disposal of electronic devices in general, and mobile phones in particular in Dubai. These gaps should be addressed by the government of Dubai and Dubai municipality to ensure a more advanced e-waste management system. 

The UAE government should also address these gaps by formulating a multilateral e-waste regulation governing all the emirates. This is further analysed in Section 4.3. 

### 4.3. Proposal of E-Waste Legislation Framework—In the Context of the UAE

E-waste is integrated into hazardous waste since it contains toxic substances for the environment and human health.

However, e-waste management systems in many countries around the world are supported by e-waste legislations which are different from one country to the other because they address each country’s specific e-waste problems. The EU, for example, is a pioneer in issuing e-waste directives among all its member nations to regulate and manage e-waste. Through its WEEE directives, issued in 2012, the e-waste management law covers e-waste collection, recycling, reuse, and metals recovery [49]. Therefore, a holistic e-waste legislation specific to the UAE should be framed to support the current e-waste management system in the country.

Similar to the WEEE directive, the UAE e-waste legislation should define e-waste clearly and ensure that this concept is understood by all stockholders. E-waste categories should all be covered in the proposed legislation, and hazardous and non-hazardous e-waste should be distinguished where the procedure of to treat the fractions of e-waste containing hazardous substances should be fully assessed. The legislation should also explain the e-waste treatment process from collection and transportation requirements to e-waste treatment including materials recovery, to the final disposal, and emphasize banning e-waste illegal transboundary movement across countries. All major stockholders from consumers, importers, retailers, municipalities, the government, and recycling companies should be clearly defined and their responsibilities must be clear. Non-compliance penalties should also be stated for any e-waste offenses. It is also important to ensure that the proposed e-waste legislation is transparent, the e-waste collection targets are well identified in the legislation based on how much e-waste is generated in the UAE, and an e-waste estimation framework should be developed specific to the UAE. Finally, compliance and monitoring are both necessary to ensure that all stockholders are working effectively, in compliance with the legislation requirements, for an effective e-waste management in the country.

## 5. Conclusions

E-waste is a growing toxic waste stream, and in the Middle East, the UAE is considered a major contributor to the generation of large quantities of e-waste. However, research in this field is still very scarce in the context of the UAE, and this study helped in shedding more light on the e-waste management system in the UAE, by taking Dubai as the main case study and waste mobile phones as a key representative of the e-waste stream. A structured questionnaire type survey was conducted among households in Dubai, based on which gaps in e-waste awareness and e-waste recycling were observed. Storing discarded electronic devices in general and old mobile phones in particular was observed as the most common disposal behavior among respondents, where the e-waste recycling rate was relatively low. To further justify the ongoing increase in e-waste quantities in the UAE, waste mobile phones was estimated for the period 2021–2030 in Dubai mainly based on primary data (collected from the survey results) using the Approximation 1 method, where three scenarios were analyzed to ensure covering different possible mobile phones lifespans. Results from the three scenarios showed that waste mobile phones are expected to increase in the targeted 10 years with the population growth in Dubai. For the baseline main scenario, the current (2021) and future potential waste mobile phones by 2030 were estimated to be 150.94 tonnes and 208.53 tonnes respectively. Therefore, recycling facilities, in compliance with Dubai municipality, should put more efforts to increase the refurbishments and recycling rates over storage at home. This study results also showed that the UAE has gaps in its e-waste inventory and efforts by the government and policymakers should be made to develop an advanced e-waste inventory for recording e-waste generation from different EEE categories, and issuing e-waste legislation is also necessary to support the suggested e-waste management system. Therefore, this study also suggested the framework of e-waste legislation to be issued in the context of UAE by the concerned authorities.

This study is limited to the emirate of Dubai for a relatively small survey sample representing its population and is mainly focusing on waste mobile phones. However, the results from this survey are still considered as a good starting point since the e-waste field is still not well explored in the context of the UAE and Dubai in particular. Therefore, findings from this study may be used by decision-makers to better understand households’ perception and behaviour toward e-waste disposal and recycling intentions.

Future studies, may extend their analysis to other UAE emirates and conduct surveys on larger samples for a longer duration. Also, future studies can extend e-waste generation and awareness analysis to other EEE categories. This will help to identify more gaps in the e-waste management system in the UAE, thereby building up a better e-waste inventory for an advanced e-waste management in the UAE.

## Figures and Tables

**Table 1:** Negative health impacts associated with e-waste common toxic substances.

Substance	Source/Type of E-Waste Examples	Health Impacts
Cadmium (Cd)	Semiconductors Infrared detectors Chip resistors	Neural Damage Irreversible impacts on human health Substance accumulation on the kidneys
Lead (Pb)	Cathode ray tubes Printed circuit boards Solder	Nervous system damage Kidney damage Blood disorders Chronic impacts on human health Negative effect/damage on the children’s brain development
Barium (Ba)	Fluorescent bulbs Front panel of CRT	Risks caused by the short-term exposure to Ba: Muscle weakness Heart and liver damage Spleen damage Brain swelling
Mercury (Hg)	Printed ciruit boards Batteries Relays and switches Flat-panel display	Brain damage Kidney damage Fetuses Damage
Nickel (Ni)	Printed circuit boards Cathode ray tubes Batteries	Lung damage Decreased lung function Lung cancer Bronchitis
Polyvinyl Chloride (PVC)	Keyboards Computer housing Cabling Monitors	Respiratory problems due to the formation of hydrochloric acid when PVC is not completely combusted Immune system damage and reproductive issues due to the formation of dioxin when PVC is burned
Polychlorinated Biphenyls (PCBs)	Transformers Capacitors Condensers	Liver damage Type 2 diabetes
Brominated Flame Retardants (BFRs)	Plastic housing of electronic devices/equipment	Endocrine system function disruption

**Table 2:** Types of waste included in the federal law No.12 of 2018 on integrated waste management, UAE [21].

Federal Law	Type of Waste
Federal law No.12 of 2018 issued on 18/12/2018 corresponding to 10 Rabi’ Al-Akhir 1440 H on the integrated waste management, UAE	Municipal Solid Waste
	Sewage Waste
	Hazardous Waste
	Construction and Demolition Waste
	Industrial Waste
	Agricultural Waste
	Marine Waste
	Oil waste

**Table 3:** Survey Sections General Description.

Survey Section	Section Description
Section 1	Includes questions 1–5 which are sociodemographic questions about the consumers’ gender, age group, education level, number of persons in his/her household, and the household monthly income.
Section 2	Includes questions 6–13 about the consumers’ awareness about e-waste, its toxic elements’ negative impacts, e-waste recycling, and consumers’ behavior toward discarded electronic devices.
Section 3	Includes questions 14–18, specific to waste mobile phones, and are about mobile phones consumption by households, respondents’ mobile phones active-use duration, respondents’ disposal behavior toward discarded mobile phones, and discarded mobile phones storage quantity and duration.

**Table 4:** Secondary Data Parameters.

Secondary Data Parameter	Description
Dubai Population 2021–2030	With referring to Dubai Statistics Center, census data for Dubai population are only available up to 2021 and has reached today 3,438,185 [24]. Based on the forecasting technique, the population was estimated to reach about 4,040,810 by 2025, while in 2030, it was predicted to reach 4,749,847.
Mobile Phones Weight	When estimating waste mobile phones generation in tonnes, the average weight of a mobile phone was taken as 0.1 kg in this study. This value was applied in many e-waste studies [31,38].

**Table 5:** Waste mobile phones estimation scenarios.

Estimation Scenario	Scenario Description
Baseline Scenario	This scenario is based on the average useful lifespan duration. This duration is estimated based on the average active-use duration of mobile phones from the survey results (mean value from question 15), and is compared to mobile phones lifespan durations in other developed countries‘ e-waste case studies.
Up Scenario	The average lifespan is pessimistic in this scenario and is shorter than the average useful lifespan taken in the baseline scenario, which in turn estimates a higher generation of waste mobile phones. This can be thus considered as the worst-case scenario. This scenario is studied because with referring to a recent study by [39], mobile phones‘ lifespan is shortening over the years and is estimated as 1–2 years for mobile phones in some developed countries. A study by [40] also stated a similar assumption for mobile phones lifespan by Japanese consumers with a duration of 2.21 years.
Low Scenario	The average lifespan is more optimistic in this scenario and is longer as it is extended by adding a storage duration based on survey results (of question 18) and an estimated reuse duration from which the total lifespan definition is used. This in turn estimates a lower generation of waste mobile phones and is hence considered as the best case scenario.

**Table 6:** Sociodemographic Results.

Sociodemographic Variable	Options	Percentage
Gender	Female	55.30%
	Male	44.70%
Age Group	18–24 years old	48.30%
	25–29 years old	22.80%
	30–39 years old	14.90%
	40–49 years old	10.50%
	50–59 years old	3.50%
	60 or older years old	0%
Education Level	Primary School	0%
	High School	9.70%
	Associate Degree	2.60%
	Bachelor’s Degree	67.50%
	Master’s Degree	15.80%
	Doctorate Degree	2.60%
	Other (please specify)	1.80%
Number of Persons in Household	1	8.80%
	2	7.00%
	3	13.20%
	4	22.80%
	5	24.60%
	6	8.80%
	7	6.10%
	8	3.50%
	More than 8	5.30%
Household Monthly Income	Below AED 2000	1.80%
	Between AED 2000–AED 5000	6.10%
	Between AED 5001–AED 10,000	7.90%
	Between AED 10,001–AED 30,000	45.60%
	Between AED 30,001–AED 60,000	21.10%
	Above 60,001	17.50%

**Table 7:** Primary Data Parameters.

Primary Data Parameter	Description
Average Household Size	Based on the survey sample which represents a fraction of the Dubai population, the average size of a household is estimated as 4.47. The latest average size of households recorded in Dubai was in 2015 as 4.2 [42]. Therefore, when compared to this value, 4.47 (i.e., survey average result) is reasonable and appropriate to use in this study.
Average Mobile Phones Consumption/Household	The average number of mobile phones currently used (i.e., currently consumed) by a household was estimated from survey results based on Figure 11 as 6.43.

**Table 8:** Lifespan scenarios.

Scenario	Justification and Comparison with Other Studies	Average Lifespan
Baseline Scenario	In this scenario, the average useful lifespan was estimated based on survey results as 3.28 years with a standard deviation of 1.68. When compared to studies in some other developed countries, the total lifespan of mobile phones in a study [28] in Australia was estimated as 3.17 years. Also, another study [43] in China has considered a service lifespan of mobile phones as 3 years, and [29] study also stated that mobile phones lifespan is in the range of 2–3 years. Therefore, it is reasonable and suitable to take the resulted survey active-use duration as the average useful lifespan in this scenario.	3.28 years
Up Scenario	A shorter lifespan of 2 years was adopted as the up scenario.	2 years
Low Scenario	According to survey results, the average storage time was 3.46 years, which is considerably high when compared to other developed countries such as Switzerland and Australia in which storage duration is less than 1 year (or does not exceed 1 year) as per studies by [28,44]. To further extend the possible total lifespan, an average estimated reuse duration of 2 years was also considered in case second-hand users exist. This gives a total lifespan of 8.74 years when considering the total lifespan definition instead of the useful lifespan definition. When compared to studies in developed countries, this scenario’s lifespan duration is overestimated and not as reasonable because the total lifespan in these countries does not exceed 3 years. However, this scenario was studied to illustrate the best-case scenario when taking a long storage based on the survey sample size and a reuse duration.	8.74 years

**Table 9:** Waste mobile phones estimation for baseline, up, and low scenarios.

Year Scenario	2021	2025	2030
Baseline (units)	1,509,447	1,774,014	2,085,299
Baseline (tonnes)	150.94	177.40	208.53
Up (units)	2,475,493	2,909,383	3,419,890
Up (tonnes)	247.55	290.94	341.99
Low (units)	566,474	665,763	782,584
Low (tonnes)	56.65	66.58	78.26

## Data Availability

Specific data about the detailed survey responses from each participant are available on request from the author. The data are not publicly available due to privacy restrictions.
